# Biosecurity perspectives of equestrian competition organizers in Ontario, Canada

**DOI:** 10.3389/fvets.2025.1713303

**Published:** 2026-01-23

**Authors:** Gabrielle K. Turcotte, Terri L. O’Sullivan, Kelsey L. Spence, Charlotte B. Winder, Amy L. Greer

**Affiliations:** 1 Department of Human Health Sciences, University of Guelph, Guelph, ON, Canada; 2 Department of Population Medicine, University of Guelph Ontario Veterinary College, Guelph, ON, Canada; 3 Department of Biology, Trent University, Peterborough, ON, Canada

**Keywords:** biosecurity, epidemiology, equestrian competitions, equine, qualitative content analysis

## Abstract

**Background:**

Biosecurity plays an important role in the prevention and control of infectious disease outbreaks in the equine population. With competition organizers responsible for implementing and upholding biosecurity requirements at competitions, it is important to understand the biosecurity landscape at these locations where Ontario horses commonly travel and interact in large group settings.

**Objectives:**

The objective of this study was to describe the perspectives, challenges and experiences of competition organizers of both sanctioned and unsanctioned events in Ontario, Canada regarding implementing equine biosecurity at competitions.

**Study design:**

This study used a qualitative content analysis in order to gather data describing the lived experiences of competition organizers.

**Methods:**

Of 53 invited participants, semi-structured, individual interviews were conducted virtually with 10 Ontario competition organizers in English disciplines (Eventing, Dressage, Hunter/Jumper). A qualitative, inductive coding method was used to analyze the interview data.

**Results:**

Interviews resulted in two major categories: (i) biosecurity at competitions is a balancing act among other important considerations and (ii) there is a disconnect between groups that play key roles in biosecurity at competitions.

**Main limitations:**

The recruitment process allowed for the possibility of introducing self-selection bias where some of the participants may have an existing interest in biosecurity, which may not be representative of all competition organizers. Individual interviews as well as the design of the interview guide helped to mitigate some potential for social desirability bias.

**Conclusion:**

Biosecurity at equestrian competitions is a multifaceted issue that requires stakeholder input and buy-in to be successful. However, there is an overall willingness from competition organizers to work toward finding a path forward to improving biosecurity and maintaining equine health and welfare at competitions.

## Introduction

Biosecurity plays an important role in the prevention and control of infectious disease outbreaks in the equine population (1, 2). As a multi-billion-dollar industry, any disruptions could have significant economic repercussions (3). Movement is a potential contributing factor for the exposure and potential spread of infectious pathogens along with congregating in large group settings (4). In Ontario, one of the major reasons for equine movement and interaction in group settings is due to competition. The Canadian National Farm and Facility Level Biosecurity Standard for the Equine Sector (5) tries to help to mitigate this risk with biosecurity recommendations for equestrian competitions. However, these recommendations do not always apply for all types of competition, nor are they always mandatory, continuing the potential risk of infectious disease outbreaks at equestrian competitions.

In Ontario, competition types include national sanctioned and local unsanctioned competitions. Sanctioned competitions must follow requirements set out by the governing body of equestrian sport, Equestrian Canada (EC), to receive EC approval prior to hosting a competition (6). In 2021, Equestrian Canada updated the biosecurity rules and regulations for their sanctioned competitions in an effort to improve biosecurity at equestrian competitions due to an increase in reported equine infectious disease outbreaks (7). As of 2021, biosecurity policies for national level sanctioned competitions included mandatory vaccination for all competing horses in all divisions as well as the completion of a Biosecurity and Response Plan Self-Assessment by competition organizers that is submitted as a part of the sanctioning process (6). In contrast, unsanctioned competitions include locally organized events such as events designed to be inviting for riders new to competitive sport as well as to provide training opportunities for less experienced horses. These competitions are not subject to any biosecurity requirements put forth by Equestrian Canada, which represents an additional challenge for equine biosecurity due to the lack of oversight and regulation.

Previous research has engaged facility owners and horse owners regarding their perception of risk and biosecurity knowledge (4, 8–11) but has not involved competition organizers. With competition organizers responsible for planning and upholding biosecurity requirements at competitions, it is important to understand the biosecurity landscape at locations where Ontario horses commonly travel and interact in large group settings. This study used a qualitative research approach in order to be able to describe some of the lived experiences of competition organizers. Qualitative studies have been key for understanding ‘hidden’ or ‘hard-to-reach’ populations (38). A qualitative content analysis was chosen specifically to be able to descriptively outline the perspectives expressed by competition organizers (12, 13). Content analysis is based in critical realism, which theorizes that perceptions of reality can change depending on personal, subjective factors (14–16). This helps to explain potential motivations or reasonings for competition organizers’ decision-making process surrounding biosecurity.

The objective of this study was to describe the perspectives, challenges and experiences of competition organizers of both sanctioned and unsanctioned events in Ontario, Canada regarding implementing equine biosecurity at competitions. This will help to inform policy that can decrease barriers for uptake of biosecurity measures to mitigate outbreak risk in equine populations.

## Materials and methods

This study was approved by the University of Guelph Research Ethics Board (REB#: 21–11-001) and is reported according to Consolidated Criteria for Reporting Qualitative Research (COREQ) guidelines (17).

Data for this study was collected through semi-structured, individual interviews. This ensured that participants could speak freely without concern for judgement from peers and to minimize any peer or professional relationship tension. While an interview guide was followed (Supplementary material), questions were mostly open-ended, allowing for more personalized and thorough discussion (18). The interviews were conducted by the first author, GT, a Caucasian, female, PhD student with Tri-Council Policy Statement: Ethical Conduct for Research Involving Humans (TCPS 2: CORE) training (specific to Canada) (19). GT has been a participant in the equine industry as a competitor, but despite this participation in the equine industry, there were no personal connections between the researcher and any of the participants.

The sampling frame for this study included all competition organizers of Eventing, Dressage or Hunter/Jumper competitions from 2017 to 2021 in Ontario. Potential participants were identified using the Equestrian Canada calendar of sanctioned competitions along with convenience sampling. Potential participants were then contacted using their publicly listed email addresses as well as directly through the research team’s connections within the equine industry in Ontario. Fifty-three competition organizers had publicly available email addresses to be able to be invited to participate in the study. This included organizers of both sanctioned and unsanctioned competitions. The inclusion criteria required participants to be 18 years of age or older and to have organized an equestrian competition of one or more of the common English disciplines (Eventing, Dressage and/or Hunter/Jumper) within the past 5 years (as of January 1, 2021) in the province of Ontario, Canada.

Potential participants completed a screening questionnaire (Supplementary material) distributed using Qualtrics XM™ (Qualtrics, Provo, UT) to gather demographic information and determine eligibility prior to scheduling an interview. The screening questionnaire also provided an overview of the study to prospective participants. Along with demographic information, the screening questionnaire asked potential participants about their responsibilities as organizers and types of events they had experience organizing. Participants were aware of the research objectives and eligibility criteria prior to providing their informed consent. Interview requests were organized with potential participants within 2 weeks of their completion of the screening questionnaire.

Interviews were conducted virtually using the Microsoft Teams platform between the interviewer, GT, and participants during the period of April through September 2022. The interview guide (Supplementary material) was followed by the interviewer included topics: general understanding of biosecurity, attitudes toward biosecurity, perspectives on the new EC biosecurity requirements for sanctioned competitions, and what the future should be for biosecurity at equestrian competitions. Interview questions were developed iteratively by the research team, and subsequently pilot tested with a person familiar with the equine industry and qualitative research. Questions were edited for clarity and flow based on pilot feedback and were approved by the research team and institutional REB prior to use in participant interviews. All interviews were audio recorded, and video recordings were also obtained when internet connection allowed and based on participant preference. Interviews lasted between 30 and 60 min and audio and video recording began after participants provided informed consent. No repeat interviews were conducted. Any identifying information was redacted or changed in the interview transcripts and participant names were excluded. Data was encrypted and stored separately. No additional interviews were conducted once the interviewer felt there were minimal new perspectives shared and repetition of similar responses was observed (20, 21).

### Data analysis

The audio recordings of the interviews were initially transcribed verbatim using the Microsoft Teams transcription service. The transcripts then underwent review by the first author to ensure clarity and accuracy when compared to the recording. The interviews underwent multiple iterations of listening and reading by GT to become familiar with the data before being imported into NVivo 14^TM^ (Lumivero, Denver, CO) for coding. A qualitative content analysis using an inductive approach was used to analyze the interview data. With an inductive approach, no pre-existing codes were used to match to meaning units in the transcript data (22). Rather, a single coder (GT) assigned open codes to the data to represent topics, perspectives, and other concepts that arose from the interview data rather than (22). A single coder allowed consistency as well as further investigation into the nuance within this smaller set of participants. The inductive approach using open coding allowed for the data to be described in the iterative process of the analysis, not with existing expectations.

Using NVivo’s data management capabilities, the first iteration of open codes were then summarized and organized based on recognized similarities and overlap into a set of initial codes. These initial codes were then organized into categories and sub-categories of similar ideas and topics (23, 24). This involved an iterative analysis of the initial codes to identify repetitive codes and codes that share common ideas. The patterns identified in the sub-categories led to the generation of preliminary categories. Preliminary categories were further refined into major themes, incorporating the similar concepts and perspectives in preliminary categories. Themes were validated amongst the co-authors, which ensured the final themes clearly encompassed what was shared in the interviews (Supplementary material). Quotes from the interviews were used to illustrate the final themes and have been edited for length and clarity as indicated using square brackets.

## Results

Of the initial 53 organizers contacted, there were 18 potential participants who completed the screening questionnaire. Of the 18 completed questionnaire responses, there were 13 eligible potential participants. Eligibility was determined by the previously stated inclusion criteria. Of the 13 eligible potential participants, 10 replied to requests to complete the semi-structured interview. Participants were from all major geographic regions of Ontario and spanned all age categories 25 and older (Table 1), with most participants being female.

**Table 1:** Demographic information of interviewed competition organizers (*N* = 10) regarding their perspectives on equine biosecurity.

Demographics	Count
**Gender**	
Man	2
Woman	8
**Primary discipline**	
Dressage	5
Eventing	1
Multi-Disciplinary (dressage, eventing, hunter/jumper)	2
Hunter/Jumper	2
**Provincial region**	
North	1
Northeast	1
East	1
Central	1
South	2
West	4
**Age**	
18–25	0
26–35	1
36–45	2
46–55	2
56–65	3
65+	2

### Theme 1: biosecurity at competitions is a balancing act among other important considerations

Participants described how organizing an equestrian competition had a variety of elements to be considered. Participants shared how they are often required to manage a long list of important tasks (including biosecurity), so when making decisions, they must consider how it would impact every other element that a competition organization entails. One of the elements that participants considered was their duty toward the horses that attended their competitions. Participants reported that they had a responsibility toward the horses that attended their competitions and demonstrated genuine care about the health and welfare of the horses. Participants were cognizant of the fact that horses were only at the competition due to their humans, and as such, the people responsible for the horses were required to ensure horses’ health and safety. Participants described how they were responsible for ensuring that horses not only stayed safe and healthy at their competitions, but also that they did not expose any other horses to potential disease risks from their events.

Participants also demonstrated an understanding of how attending competitions could put horses at risk of exposure to an infectious disease. Participants mentioned a variety of risk factors for infectious disease spread including stabling, direct contact, equipment sharing, humans as a vector of transmission, and airborne transmission. However, participants also mentioned how they must also take into account other tasks that they consider to be of equal or even greater priority. For example, there were concerns regarding parking, course design and facility maintenance, which organizers have stated to be more visible and of immediate concern due to their imminent safety and reputational implications. On the other hand, participants found that potential areas of risk for infectious disease spread were more challenging to visualize. This was compounded by the fact that many participants shared that they had not had a recent experience with an infectious disease outbreak at equestrian competitions, which lowered biosecurity on an organizers’ list of priorities.

Participants raised financial concerns in association with implementing biosecurity measures, since they recognized that competitions are businesses. They described how any costs associated with biosecurity would not only be incurred by competition organizers, but also competitors, which will then secondarily also impact competitions. There was a concern from participants that equestrian facilities that would be mandated by competitions to vaccinate all of their horses will either choose to no longer attend competitions or choose to compete elsewhere due in part to the additional costs of vaccination. Participants also described the impact of anticipated increasing costs of human labour, competition venue upgrades, and supplies with the implementation of additional biosecurity requirements. For example, participants shared that many of the current stabling areas allow for nose-to-nose contact over the top of the stall walls. Participants were aware that this was a biosecurity risk and mentioned preferring to see walls that are high enough with no openings to prevent contact. However, the reported concern from some participants was that some of the major venues have hundreds of stalls that would need to be changed, costing a substantial amount of money that neither the competitions nor the venues have available to spend. Participants also said that the final decision for any renovation to the stabling areas would be on the venue, no matter what the organizers’ wants or needs. Alternative to renovation, participants mentioned the option of spreading out the groups of horses participating in the competition, so there was no direct contact between horses from different stabling groups. While this would improve the biosecurity at equestrian competitions, the concern for some participants was that space at some competition venues is limited. It was explained that this may make it impossible to accommodate the same number of competitors and thus accrue the same revenue if organizers should become required to spread out the groups.

Participants also reported their concern that increased biosecurity measures may result in their shows occurring on a much smaller scale, less frequently, or not at all should the number of entries decrease due to the financial ramifications of biosecurity. Participants demonstrated concern that these additional costs may become a financial barrier to competition that they themselves would not want to create for their competitors. This was especially important when discussing continuing to promote the grassroots levels of competition to try to grow an already costly sport.

Time constraints were also a concern of participants. Participants reported that any of the tasks associated with ensuring all biosecurity regulations were being followed took up additional time that they already consider to be limited when trying to organize competitions. Some of the responsibilities mentioned included follow-ups on incomplete or missing information from competitors, and additional site visits to the competition venue prior to the competition to complete the required documentation in its entirety. Participants stated that these are tasks often undertaken by themselves as they considered additional staff to be a cost they may not be able to afford or not be able to find. Participants expressed appreciation for the volunteers they are reliant upon to ensure competitions are able to run smoothly but already found them to be difficult to find in the numbers they would like. Overall, to accomplish and enforce the degree of biosecurity that participants know to be of the current highest standard, they believed they would need more time, money, and many more people (Table 2).

**Table 2:** Selected participant quotes for each theme.

Theme	Select participant quotations
Biosecurity at competitions is a balancing act among other important considerations	*“[Biosecurity is not] the top of my priority list as a horse show manager. I’ve never had any bad experiences, so it’s hard to get too concerned about it.” – INTERVIEW 5*
	*“If you make them all get their $100 vaccines, they are not gonna bring their horses to the show anymore.” – INTERVIEW 1*
	*“I certainly have thought about many things, but I find it’s difficult to implement them. Being a smaller show, I find we struggle to get enough help with things.” – INTERVIEW 10*
There is a disconnect between groups that play key roles in biosecurity at competitions.	*“I think everyone has to be on board with [biosecurity] because it does not work otherwise. So, I think as show organizers we have certain things everyone needs to provide. But I think it’s also the responsibility of competitors to be safe, to take temperatures, to be diligent with that.” – INTERVIEW 7*
	*“I think there’s a level of understanding of what spreading disease is about […], but then in the same breath, [competitors] go and use a rub rag on three different horses.” – INTERVIEW 3*
	*“There were a couple of changes I made to our document based on the self-assessment, but there’s stuff in there that made no sense for a horse show.” – INTERVIEW 9*

### Theme 2: there is a disconnect between groups that play key roles in biosecurity at competitions

With new biosecurity measures placing additional responsibility on competition organizers, participants expressed that while they held some responsibility for biosecurity, it should also be shared with other key groups at competitions. Participants mentioned horse owners, riders, and trainers, as some of the other parties that they at times considered to not always be doing their part when it came to biosecurity. Based on the participants’ shared experience, there were limits to how far competition organizers could go to ensure a certain level of biosecurity was being followed at competitions. It was then on other key parties associated with competition horses to participate as well. Participants described this to be particularly important since they believed there was no real way to enforce biosecurity at competitions. Participants also described how they believed they did not have the authority to try and penalize anyone for not following biosecurity protocols.

Participants also expressed their confusion regarding the exact consequences for potential biosecurity non-compliance issues and who was responsible for the delivery of potential consequences. Participants described how they tried their best to ensure all the regulations were followed, but ultimately, participants considered the owner or rider to be the person responsible for their horse and its actions.

This ties into participants’ perception of their patrons which is that there is a gap between competition organizers’ level of education and prioritization of biosecurity and that of their competitors. Participants shared that while they themselves may know about biosecurity and what it entails, those attending their competitions were either less educated about biosecurity or did not place importance on it. This assessment was based on behaviors some participants had observed at competitions, such as allowing for grazing, interactions between horses from different facilities, and equipment sharing.

Participants also expressed that they were not only responsible for putting forth policies to protect the horses but also educate their competitors. Some participants had even described how they had taken it upon themselves to distribute biosecurity information sheets or provide explanations for specific biosecurity measures they had chosen to add to their competitions. Participants describe their feeling that these efforts were often futile as they perceived they were sharing this information with an audience that did not have a desire to engage with the materials.

While participants stated how they believed they could ask their competitors to follow their guidelines and read certain documents prior to attending their competition, it was also participants’ belief that it was up to the person responsible for the horse to decide how important they considered this information to be. They also believed that the person responsible for the horse should also ensure that the support team for their horse is aware of the expectations when it came to biosecurity. This team could include grooms, friends, family, braiders, farriers, and vets. Participants also described how this responsibility could be extended to trainers and coaches who are able to set the tone and educate their students on the importance of biosecurity and what it entails at competitions.

Participants also shared the challenges they faced in implementing the biosecurity requirements placed upon organizers by their sanctioning body. Some elements included in the documentation process came across to organizers as infeasible for the reality of equestrian competitions. Participants expressed that at times what was being asked of them was not relevant or possible for their particular competition format or process. The current guidelines and recommendations came across to participants as generalized for all competitions. Many participants shared that they felt like the requirements for their competitions were unrealistic and did not account for the reality of the diversity of competitions in Ontario. Participants expressed a desire for biosecurity recommendations to be tailored to their competition’s characteristics and structure. They believed that competitions with 10–20 horses for a half day with little to no stabling should not be expected to implement the same biosecurity protocols as a competition with hundreds of horses over multiple days with stabling. Participants stated that they often used their own discretion to adapt the biosecurity recommendations for their competition. Participants shared that that smaller competitions were often forgotten about or were required to put in even more effort to keep up with the requirements for biosecurity from governing bodies. This led participants to describe how this made them feel frustrated, confused, and, at times, fearful when it came to the relationship with those responsible for sanctioning their competitions and instituting biosecurity regulations.

Part of participants’ fear was described to be attributed to the perceived lack of communication of important information. Some participants were not aware of the new requirement as of January 1, 2021, of an Equine and Biosecurity and Response Plan Self-Assessment, which led them to feel like they were “scrambling at the last minute” to complete all of the necessary categories by the sanctioning deadline. For participants that were aware of the document, there was also a perceived lack of clarity and guidance on how this documentation was to be best completed. Some spoke with colleagues, veterinarians or consulted the internet in order to be able to complete the document in time. Many shared that they could have used additional information, especially from the governing body who required the completion of the document. There was concern that the poor completion of this document would result in not being able to run the sanctioned competition that participants’ competitors expect.

Participants expressed that there were few places to turn that were able to effectively and efficiently provide the information they required. Participants shared that there is a seeming lack of summarized documentation that is both understandable and shareable as needed. This included documentation on vaccination, disease prevention, how infectious diseases spread, or current outbreak data. Participants also mentioned that they were often left sifting through different sources of information to try and determine the best course of action for their particular set of needs.

## Discussion

This research highlights that the competition organizers interviewed for this study care for competition horses’ health and welfare, and demonstrated evidence of knowledge surrounding what biosecurity entails. However, they are faced with considerable challenges to balance that demotivate them from participating in biosecurity to a higher degree despite best intentions. These challenges include financial considerations, time constraints, lack of clarity on enforcement and responsibility, and a disconnect between competitors, their patrons and the sanctioning body for equestrian competitions.

While this study interviewed only a portion of all competition organizers, the qualitative study design allowed for the opportunity for a more in-depth discussion of the motivation and rationale for participant perspectives. The smaller number of participants was also reflected in the limited number of individuals representing competitions in each English equestrian discipline. The range of disciplines represented was still important and similar perspectives were still obtained across disciplines, indicating a potential for this to be applicable for the broader competition organizer population in similar disciplines in Ontario. Part of the explanation for this cross-discipline similarity could also be attributed in part to the recruitment process. This process allowed for the possibility of introducing self-selection bias where some of the participants may have an existing interest in biosecurity, which may not be representative of all organizers.

Qualitative research also can be limited due to the potential for social desirability bias from participants among their peers Conducting interviews individually and confidentially as well as the design of the interview guide helped to mitigate some of this potential for social desirability bias (25). Participants were mostly female, but this is known to those familiar with the equine industry to be representative of equestrian sport in English disciplines (26), which was the focus of this study. The smaller, more homogeneous population is why we believe data saturation (when no new themes emerge from responses) was reached (20). The consistency of responses was also further ensured due to the use of a single coder. While this may negatively affect internal validity, this was not the goal of the study. As a preliminary investigation, the described challenges stated by interviewees provide a baseline as to what must be understood and addressed to better guide future biosecurity recommendations at equestrian competitions in English disciplines.

The perspectives shared by competition organizers interviewed echo the results of studies in other similar stakeholder groups in the equine industry when it comes to biosecurity. Veterinarians, horse owners and facility managers, among other groups in the equestrian competition environment, also face similar, conflicting challenges in prioritization when it comes to biosecurity. This can be in part due to horse owners’ belief that some risk is inevitable and there was no way to completely protect a horse from infectious disease, therefore prioritization of biosecurity can feel futile (11). Similarly, research among horse owners demonstrated that they believe the horses are their responsibility, but tend to leave biosecurity up to the facility where their horse is stabled (11). Similar to competition organizers, facility managers also shared facility-related and time-related challenges when trying to implement biosecurity measures to keep horses in their care healthy and safe (4, 39). These findings demonstrate that different stakeholder groups are faced with similar competing priorities when it comes to engaging in equine biosecurity while also having to manage relationships with other key parties involved in equine health and welfare (27). This commonality among stakeholder concerns provides an avenue for potential improvement in the uptake of biosecurity recommendations by addressing these shared challenges.

The disconnect described by competition organizers has also been demonstrated in veterinarians. Vets expressed a sense of disconnect from their clients, and horse owners, and also questioned some of the practicality of biosecurity measures compared to their theoretical use (28). This disconnect between veterinarians and clients could be attributed to veterinarians’ perception of their clients who appear to rely on veterinarians to provide up to date biosecurity information as competition organizers felt about their competitors. Similarly to competitors, clients also have varying degrees of knowledge of infectious diseases causing differing expectations from veterinarians surrounding biosecurity information to be shared and aware of (28). This was stated to be in part due to the fact that owners feel that they need support from other groups such as veterinarians in order to make the correct biosecurity choices and execute them, as what is provided by the government is not always considered to be practical (11). This demonstrates a common sentiment of a lack of clarity in communication and expectations across multiple stakeholder groups, including competition organizers, in the equine industry.

The complex relationships between organizers, competitors, and sanctioning bodies appeared to also contribute to the low and inconsistent degree of motivation to participate in biosecurity. Organizers must ensure the requirements from sanctioning bodies are met, but it is challenging to try and continue to improve the level of biosecurity when some organizers stated that any changes would not be received by their clients in a way that would be productive or efficient. There is also the potential for pushback from clients should biosecurity result in increased costs for them to compete. It has been expressed in interviews that these are financial risks that competition organizers may not be able to afford. These increased costs associated with biosecurity improvements could include potential additional staffing, supplies and facility upgrades. This is also particularly challenging when competition organizers already feel like they are faced with considerable costs and additional pressing priorities to be able to successfully run their competitions. The potential for negative feedback or decline in competition participation may contribute to the perceptions of biosecurity requirements being infeasible or not relevant for competitions as well as the issues surrounding motivation. To address these challenges, all parties must come together and work toward a common goal for individuals to participate in biosecurity on a consistent basis and to alleviate the concern of decreasing numbers of competitors.

To try and overcome some of the perceived disconnect among stakeholder groups, one of the pathways toward improved biosecurity at equestrian competitions could incorporate a teamwork approach. A teamwork approach has been associated with increased motivation in the workplace and could be extended to competitions as this is also a workplace for competition organizers as well as some competitors (29). This would allow for individuals to come together and work toward a common goal. There is also evidence that health outcomes can be improved when individuals are more engaged in the decision-making process surrounding their health (30). This could be applied to competitor and organizer engagement with governing bodies and policy makers in the teamwork approach. Should these stakeholder groups feel as if they have more of a say in the biosecurity policy decision making process, there is a chance for better outcomes in biosecurity implementation.

At the same time, the motivational challenge of biosecurity is also compounded by the fact that biosecurity’s success is measured by non-events. When no outbreaks occur, this can lead some to believe that biosecurity measures are moving in the correct direction, when it may be simply due to chance. However, when no outbreaks occur, it is challenging to make the argument that biosecurity measures should continue to be used and improved when it is not considered to be a pressing issue at equestrian competitions. This is an example of the impact of optimism bias in human decision-making. Humans can have a tendency toward assuming the best instead of facing the uncertainty and unprecedented nature of catastrophic situations including infectious disease outbreaks (31, 32). Therefore, while the risk is understood, if it has not been a notable problem for competitions in recent memory, outbreaks become an almost an unimaginable occurrence. Therefore, stakeholders should be provided continued educational opportunities to remain aware of current outbreaks, ways outbreaks can and have been mitigated, and the potential catastrophic scope of what could occur if biosecurity is not prioritized (33, 34).

Another pathway to improve competition biosecurity could be the creation of a more individualized biosecurity protocol for the variety of equestrian competitions that occur. While this is not currently common practice in the equine industry, in the beef cattle industry, the use of semi-individualized biosecurity protocols has been a tool used to overcome challenges associated with the variation in facilities across the beef cattle industry (35). Preliminary evidence has shown that tailored, veterinary-led biosecurity advice has the potential for pathogen spread reduction when compared with traditional biosecurity measures (35). This approach is particularly promising when paired with individual-level on-farm training on hygiene procedures (36). It is also common practice in human health for individuals to assess their level of risk and adjust the degree to which they engage with protective behaviors accordingly, as seen with COVID-19 (37). Additionally, success in other livestock groups such as cattle and swine can be used to demonstrate the potential for feasibility of biosecurity measures at equine facilities with similar challenges. Further investigation into the design of semi-individualized biosecurity protocols specific to equestrian facilities and competition would be a key next step in biosecurity improvements for the equine industry.

In conclusion, the results of this content analysis demonstrate that biosecurity at equestrian competitions is a multifaceted issue that requires stakeholder input and buy-in to be successful. There is a perceived division between stakeholder groups in terms of communication and ideology when it comes to biosecurity. However, there is an overall willingness from competition organizers to work toward finding a path forward to improving biosecurity and maintaining equine health and welfare at competitions. Designing biosecurity requirements for competitions moving forward should acknowledge the challenges faced by competition organizers and understand their needs. A semi-individualized biosecurity approach that is able to be tailored to specific needs and situations should be investigated in the equine population as a potential next step toward improving biosecurity uptake for those involved in equestrian competitions. Humans hold the decision-making power when it comes to horses, therefore, it is important to try to motivate key stakeholders to work together toward the common goal of maintaining equine health and welfare.

## Data Availability

The raw data supporting the conclusions of this article will be made available by the authors, without undue reservation.
