# Supplementary material for: Biosecurity perspectives of equestrian competition organizers in Ontario, Canada
Source: Front Vet Sci. 2026 Jan 23;12:1713303. doi: 10.3389/fvets.2025.1713303 (PMC12875910; doi:10.3389/fvets.2025.1713303)
Supplement: Supplementary file 1 [file Data_Sheet_1.pdf]

Supplementary Material

Screening Questionnaire

Start of Block: Eligibility

I would like to begin by thanking you for taking the time to complete this questionnaire. We are investigating the specific factors and perceptions that competition organizers consider to be most influential when it comes to the implementation of biosecurity protocols recommended by Equestrian Canada.

All responses will be kept confidential. This means that your responses to the questions in this questionnaire will only be shared with the research team and any findings included in the report will not identify you as the respondent. You do not have to discuss anything you do not feel comfortable with and may choose to exit the questionnaire and revoke your consent to participate in this study at any time. Should you agree to participate in the research interviews but choose to skip specific questions or end the interview at any point, you will still be eligible for the gift card.

This questionnaire should take approximately 5-10 minutes.

End of Block: Eligibility

Start of Block: Consent

Do you consent to participate in this study?

- o Yes
- o No

End of Block: Consent

Start of Block: Demographics

Which of the categories below includes your age?

- o 18-25
- o 26-35
- o 36-45
- o 46-55
- o 56-65
- o 66+
- o prefer not to say

What is the highest level of education you have completed?

- o High School Diploma
- o College Diploma
- o University Degree (ie. Bachelor of Science, Bachelor of Arts etc.)
- o Graduate Degree (ie. Masters' degree, PhD etc.)
- o Professional Degree (ie. DVM etc.)
- o Other: ____
- o prefer not to say

What is your gender?

- o Man
- o Woman
- o My gender is not listed above:____________________
- o Choose not to respond

End of Block: Demographics

Start of Block: Experience

Please describe your **current role(s)** as an event organizer?
(ie. Manager, Show Secretary, Treasurer, Steward etc.)

____

What are some of the responsibilities and tasks you have completed in your role(s) as an equestrian event organizer? Be as specific as you can.

____

____

How long have you been in your current role(s) that you described in the previous questions?

- o Less than 5 years
- o 5-10 years
- o 11-20 years
- o 21+ years

Which level(s) of sanctioned events have you organized?
(choose all that apply)

- o Unsanctioned
- o Bronze

- Silver
- Gold

For which equestrian disciplines have you organized an event? (select all that apply)

- Dressage
- Eventing
- Hunter/Jumper
- Western
- Endurance
- Other: ____

Approximately how many of each event type selected above have you organized in the past 5 years?

____

____

____

____

____

End of Block: Experience

Start of Block: Interview

Please provide your contact information below to schedule your follow-up interview.

- Email: ____
- Cell Phone: ____
- Home Phone: ____

Do you have access to a strong Wi-Fi connection for the purpose of participating in a video interview using a video conferencing platform (ie. Zoom, Microsoft Teams etc.)?

- Yes
- No
- Sometimes

Please rank your preferred contact method

______ Home Phone

______ Cell Phone

_______ Email

_______ Text Message

_______ Social Media (insert handle in text box if applicable)

Do you have a preferred time to be contacted?

- o Weekdays
- o Weeknights
- o Weekends
- o Other: ____

Would you like to be contacted at the end of the study to receive a copy of the findings?

- o Yes
- o No

End of Block: Interview

Interview Guide

Introduction and Consent

First, I would like to begin by thanking you for taking the time to meet with me today. My name is Gabrielle Turcotte, and I am a PhD student from the Ontario Veterinary College at the University of Guelph. I would like to talk to you about your experiences in organizing equestrian competitions in Ontario. Specifically, we are looking to investigate the specific factors and perceptions that competition organizers consider to be most influential when it comes to the implementation of biosecurity protocols at equine events.

This interview should take about 45-60 minutes and I will be recording the session I don't miss any of your comments. While some notes will also be taken during the session, I am not quite fast enough to get everything down. If you have any trouble hearing me or if I am unable to hear you clearly, some sections may be repeated in order to ensure nothing is missed in the recording as well.

Additionally, all responses will be kept confidential. This means that your responses to the questions in this interview will only be shared with the research team and any findings included in the report will not identify you as the respondent. You do not have to discuss anything you do not feel comfortable with, and you may end the interview at any time.

Do you have any questions about the process? Is there anything you would like to share before we begin?

Do I have your consent to participate in this interview?

Introduction Questions

i. Tell me about how you first got into the competition world? When was your first competition?
ii. When was the first time you organized a competition?

Section 1: Equine Biosecurity Understanding (approximately 5-8 minutes)

I want to start a conversation to help me understand what you think about and what you know about the term biosecurity and how it relates to horses.

1. In your own words, can you explain what you believe biosecurity means?

2. Based on your description of biosecurity, how do you believe it is perceived in the equine industry?

- Follow-up questions (if necessary):

- Why do you believe that is?
- In what capacity do you think it should play a role?
- At what level? (farm, competition, international etc.)
- Why do you believe this? Or why not?

3.In your experience with organizing equine events, how was equine biosecurity taken into consideration when planning your events?

- Follow-up questions (if necessary):
    - Do you think it should be? To what degree and why?
    - Has this changed in recent years? Why do you think so?
    - Explain some of the changes that have been positive or negative from your perspective

4. Elaborate on the level of understanding you believe your patrons (owners/riders) have of what equine biosecurity is and what is required of an event such as yours to ensure horse safety and welfare at shows?

5. Based on your answer to my previous questions, do you believe there are some expectations patrons (owners/riders) have related to biosecurity at events?

- Follow-up questions (if necessary):
    - What do you think these expectations may be?
    - Why do you believe these exist?
    - How do you feel about these expectations?

6. Biosecurity is a complex, multifaceted issue affecting many different groups of individuals. Why do you believe it matters? Or does it?

7. Who do you believe is responsible for biosecurity when it comes to maintaining animal health and welfare when attending events?

- Follow-up questions (if necessary):
    - Is it Owners? Riders?
    - Venue Owners/Event Organizers?
    - Spectators? Governing Bodies?
    - Why do you believe it is their responsibility?

Section 2: Attitudes towards Regulations (15-20 minutes approx.)

2021 was the first year of the new Equine Biosecurity and Response Plan Self-Assessment so I would like to talk to you about your experience with it.

The following section applies to those who answered YES in the pre-interview questionnaire when asked if they participated in organizing an Equestrian Canada

sanctioned equestrian competition between January 1, 2021, and December 31, 2021. If the answer was NO respondents will proceed straight to Section 2b.

Section 2a: Equine Biosecurity and Response Plan Self-Assessment

8. How do you feel about the new EC requirement for the completion of an Equine Biosecurity and Response Plan Self-Assessment for all EC sanctioned competitions?

- Follow-up questions (if necessary):
    - Are there some positive elements? Some negative elements?
    - Why do you believe it was implemented?
    - Do you feel like there has been a change in patrons' perception as of late, your liability etc.?

9. Did this new regulation impact your perspective on how to prepare for and run a competition?

- Follow-up questions (if necessary):
    - If yes, how?
    - If no, why?

10. How would you describe the process of fulfilling the equine biosecurity requirements? What is involved? How does it rank on a scale of 1 (easy) to 5 (very challenging)?

- Follow-up questions (if necessary):
    - Why do you rank it as…?
    - What were some of your most difficult challenges relating to equine biosecurity this year? (aside from COVID-19)
    - What made them particularly challenging?

11. When developing your biosecurity and response plan, who do you go to for up-to-date biosecurity information and/or support?

- Follow-up questions (if necessary):
    - Why do you trust them to give you the correct information?
    - Is there anyone you do not trust? Why?

12. Are there specific additional costs associated with these additional requirements? For example, were additional time or labour costs incurred?

- Follow-up questions (if necessary):
    - Approximately how much?
    - How did you manage to deal with these added costs? Were they absorbed? Transferred to patrons etc.?

- Do you believe these costs are worth the investment in biosecurity?

13. What is your perception of the effectiveness of the current Equine Biosecurity and Response Plan Self-Assessment and regulations on equine events, is it an effective tool to improve equine health and welfare?

- Follow-up questions (if necessary):
  - Why is it effective? What elements are?
  - If not effective why? What do you believe is missing?

<u>Section 2b: Long-Term Impact</u>

14. In your experience, could you explain what the current level of biosecurity is for horses attending competitions across Ontario?

- Follow-up questions (if necessary):
  - Is there anything you think should be improved or anything that you believe works particularly well to ensure optimal equine health and welfare?
  - Why do you think it works? Why doesn't it work?

15. What do you think about the long-term impact or staying power related to some of the changes that have been made thus far regarding competition events and the requirements for organizers in terms of biosecurity?

- Follow-up questions (if necessary):
  - Is this something you believe should continue?
  - What do you think would improve the ease of implementation year after year?
  - Why do you believe these changes are important (or not) for your role in organizing these events?

Biosecurity requirements (The EC biosecurity and response plan) are only required for sanctioned shows, therefore we'd also like to discuss the comparison between sanctioned and unsanctioned competitions.

16. What is your perception of the differences between organizing an unsanctioned vs sanctioned event?

- Follow-up questions (if necessary):
  - What do you believe are some advantages and disadvantages to both?
  - Why do you believe some may choose to organize or attend sanctioned vs. unsanctioned events?

17. Do you believe those differences are reasonable between regulations for sanctioned and unsanctioned events, do they seem logical? Why or why not?

Section 3: Next Steps (10 minutes approx.)

18. What would you like to see improved in the implementation process of biosecurity protocols from an organizer perspective?

- Follow-up questions (if necessary):
  - Why? How would these be of help to you?

19. How could biosecurity processes for equestrian competitions be designed to be easily implemented on a longer-term basis for organizers?

- Follow-up questions (if necessary):
  - Why does this help organizers?
  - Who else may be positively (or negatively) impacted by this design?

20. What impact, if any, do you think the EC biosecurity and response plan will have on Ontario equestrian competitions as a whole?

- Follow-up questions (if necessary):
  - Do you think there is a possibility of some competitions moving to becoming unsanctioned? Why do you think so?

21. In your own words, describe what you believe would be an ideal process for implementing biosecurity measures to ensure health and safety of equine athletes in Ontario when attending competitions?

- Follow-up questions (if necessary):
  - Why is this the ideal process for you?
  - How do you think it would help make the measures more successful?

22. If you had one recommendation to make to improve equine biosecurity regulations, what would it be and why?

Section 4: Closing Remarks (5 minutes approx.)

23. Do you have any other thoughts you would like to share about equine biosecurity and the new biosecurity and response plan requirement?

24. Is there anything you would like to share that we have not yet discussed?

Thank you very much for your time. I will be analyzing the responses in the next couple of months and am happy to share with you a copy of the draft when it is completed if you are interested. Thank you again and have a lovely rest of your day.

Figure S.1 Participation flow diagram for recruitment process

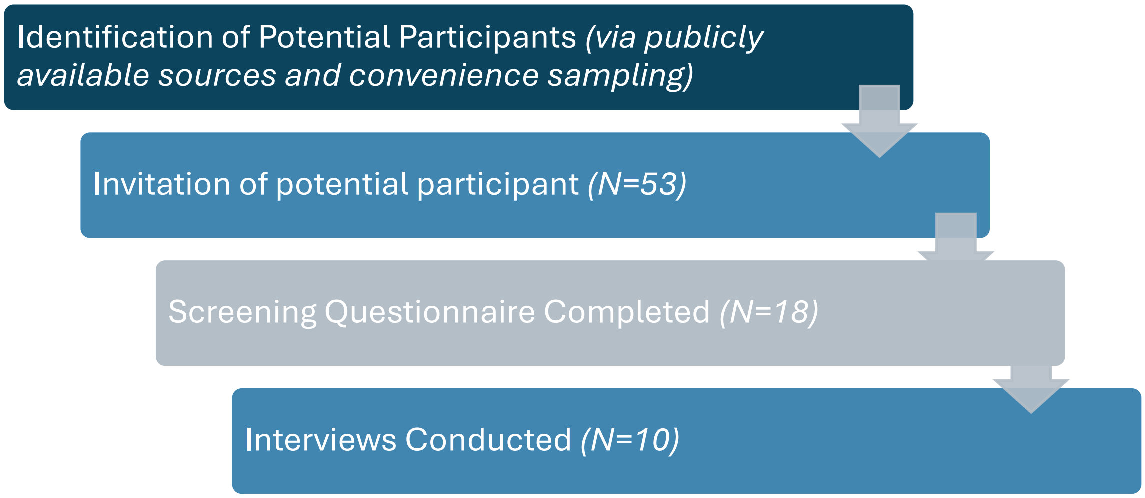

Table S.1: Codes, sub-categories, categories and themes from content analysis of ten equestrian competition organizer interviews in Ontario, Canada

Code	Sub-category	Category	Theme
Additional comfortability in competition attendance	Personal choice	Biosecurity motivations	**Biosecurity at competitions is a balancing act among other important considerations.**
Additional work for vets	Labour	Biosecurity drawbacks	**Biosecurity at competitions is a balancing act among other important considerations.**
Administrating existing behaviour	Labour	Out of touch	**There is a disconnect between groups that play key roles in biosecurity at competitions.**
Announcing Biosecurity understanding to keep people apprised	Sharing Information	Communication gaps	**There is a disconnect between groups that play key roles in biosecurity at competitions.**
Ability of biosecurity documentation as a form of education	Education	Education	**There is a disconnect between groups that play key roles in biosecurity at competitions.**
Access to information and ease of finding it	Sharing Information	Communication gaps	**There is a disconnect between groups that play key roles in biosecurity at competitions.**
Additional Information recommended	Education	Education	**There is a disconnect between groups that play key roles in biosecurity at competitions.**
Briefness of process completing biosecurity documentation	Not a challenge	Biosecurity motivations	**Biosecurity at competitions is a balancing act among other important considerations.**
Communication issues and improvements for better clarity	Sharing Information	Communication gaps	**There is a disconnect between groups that play key roles in biosecurity at competitions.**

Desire for documentation to be able to be more individualized	Individualization	Personalized approach	**Biosecurity at competitions is a balancing act among other important considerations.**
Fear surrounding ability to complete what is required	Emotional Toll	Communication gaps	**There is a disconnect between groups that play key roles in biosecurity at competitions.**
Follow ups on incomplete documentation	Time constraints	Biosecurity drawbacks	**Biosecurity at competitions is a balancing act among other important considerations.**
Frustrating elements involved in biosecurity documentation	Emotional Toll	Biosecurity drawbacks	**Biosecurity at competitions is a balancing act among other important considerations.**
Necessary for biosecurity	Not a challenge	Biosecurity motivations	**Biosecurity at competitions is a balancing act among other important considerations.**
Need for increased efficiency	Individualization	Biosecurity drawbacks	**Biosecurity at competitions is a balancing act among other important considerations.**
Not costly	Not a challenge	Biosecurity motivations	**Biosecurity at competitions is a balancing act among other important considerations.**
Out of touch with reality	Irrelevant	Out of touch	**There is a disconnect between groups that play key roles in biosecurity at competitions.**
Presence of repetition and redundancies in biosecurity documentation	Irrelevant	Out of touch	**There is a disconnect between groups that play key roles in biosecurity at competitions.**
Time Consuming	Time constraints	Biosecurity drawbacks	**Biosecurity at competitions is a balancing act among other important considerations.**

Unawareness of biosecurity mandates for competitions	Irrelevant	Communication gap	**There is a disconnect between groups that play key roles in biosecurity at competitions.**
Unknown information but required to complete documentation	Irrelevant	Communication gap	**There is a disconnect between groups that play key roles in biosecurity at competitions.**
Use of required documentation as an effective tool for biosecurity improvement	Education	Education	**There is a disconnect between groups that play key roles in biosecurity at competitions.**
Biosecurity is irrelevant at competitions	Irrelevant	Biosecurity is low priority	**Biosecurity at competitions is a balancing act among other important considerations.**
Biosecurity is not a major concern	Irrelevant	Biosecurity is low priority	**Biosecurity at competitions is a balancing act among other important considerations.**
Biosecurity should be able to adapt depending on the situation	Individualization	Personalized approach	**There is a disconnect between groups that play key roles in biosecurity at competitions.**
Increase further in future	Biosecurity is important	Future expectations	**There is a disconnect between groups that play key roles in biosecurity at competitions.**
Infeasibility of biosecurity improvements	Irrelevant	Out of touch	**There is a disconnect between groups that play key roles in biosecurity at competitions.**
Negative Change	Irrelevant	Future expectations	**There is a disconnect between groups that play key roles in biosecurity at competitions.**
No changes observed nor possible or necessary	Irrelevant	Future expectations	**There is a disconnect between groups that play key roles in biosecurity at competitions.**

Positive Change	Not a challenge	Future expectations	**There is a disconnect between groups that play key roles in biosecurity at competitions.**
Will continue in the same fashion	Status quo	Future expectations	**There is a disconnect between groups that play key roles in biosecurity at competitions.**
Cleaning	Biosecurity understanding	Biosecurity measures	**Biosecurity at competitions is a balancing act among other important considerations.**
Cohorting	Biosecurity understanding	Biosecurity measures	**Biosecurity at competitions is a balancing act among other important considerations.**
Contact tracing	Biosecurity understanding	Biosecurity measures	**Biosecurity at competitions is a balancing act among other important considerations.**
Direct contact	Biosecurity understanding	Competition risk	**Biosecurity at competitions is a balancing act among other important considerations.**
Disconnect from biosecurity on a personal level	Biosecurity understanding	Biosecurity is low priority	**Biosecurity at competitions is a balancing act among other important considerations.**
Equipment Sharing	Biosecurity understanding	Competition risk	**Biosecurity at competitions is a balancing act among other important considerations.**
Flow of Traffic	Biosecurity understanding	Biosecurity measures	**Biosecurity at competitions is a balancing act among other important considerations.**
Foot bath	Biosecurity understanding	Biosecurity measures	**Biosecurity at competitions is a balancing act among other important considerations.**

Grazing	Biosecurity understanding	Competition risk	**Biosecurity at competitions is a balancing act among other important considerations.**
Health and welfare of horses	Biosecurity understanding	Biosecurity motivations	**Biosecurity at competitions is a balancing act among other important considerations.**
Humans as Vector of transmission	Biosecurity understanding	Competition risk	**Biosecurity at competitions is a balancing act among other important considerations.**
Invisible hazard	Biosecurity understanding	Biosecurity drawbacks	**Biosecurity at competitions is a balancing act among other important considerations.**
Lack of understanding	Biosecurity understanding	Education	**There is a disconnect between groups that play key roles in biosecurity at competitions.**
Large Group Setting	Biosecurity understanding	Competition risk	**Biosecurity at competitions is a balancing act among other important considerations.**
Manure disposal	Biosecurity understanding	Competition risk	**Biosecurity at competitions is a balancing act among other important considerations.**
Monitoring	Biosecurity understanding	Biosecurity measures	**Biosecurity at competitions is a balancing act among other important considerations.**
Movement	Biosecurity understanding	Competition risk	**Biosecurity at competitions is a balancing act among other important considerations.**
Not bringing sick horses to competition	Biosecurity understanding	Biosecurity measures	**Biosecurity at competitions is a balancing act among other important considerations.**

Only matters at the highest level	Biosecurity understanding	Biosecurity is low priority	**Biosecurity at competitions is a balancing act among other important considerations.**
Only of importance when there is an outbreak	Biosecurity understanding	Biosecurity is low priority	**Biosecurity at competitions is a balancing act among other important considerations.**
Outbreaks as a disruption for competitors	Biosecurity understanding	Biosecurity motivations	**Biosecurity at competitions is a balancing act among other important considerations.**
Particle spread	Biosecurity understanding	Competition risk	**Biosecurity at competitions is a balancing act among other important considerations.**
Potential Negative effects	Biosecurity understanding	Biosecurity motivations	**Biosecurity at competitions is a balancing act among other important considerations.**
Protection of the horse	Biosecurity understanding	Biosecurity motivations	**Biosecurity at competitions is a balancing act among other important considerations.**
Quarantine	Biosecurity understanding	Biosecurity measures	**Biosecurity at competitions is a balancing act among other important considerations.**
Reference to livestock	Biosecurity understanding	Communication gaps	**There is a disconnect between groups that play key roles in biosecurity at competitions.**
Stabling	Biosecurity understanding	Competition risk	**Biosecurity at competitions is a balancing act among other important considerations.**
Temperature checks	Biosecurity understanding	Biosecurity measures	**Biosecurity at competitions is a balancing act among other important considerations.**

Theory vs Practice	Biosecurity understanding	Biosecurity drawbacks	**Biosecurity at competitions is a balancing act among other important considerations.**
Transportation	Biosecurity understanding	Competition risk	**Biosecurity at competitions is a balancing act among other important considerations.**
Trust	Biosecurity understanding	Biosecurity measures	**Biosecurity at competitions is a balancing act among other important considerations.**
Vaccination	Biosecurity understanding	Biosecurity measures	**Biosecurity at competitions is a balancing act among other important considerations.**
Biosecurity is only as good as its weakest link	Biosecurity understanding	Biosecurity drawbacks	**Biosecurity at competitions is a balancing act among other important considerations.**
Biosecurity is somewhat a foreign concept to people	Biosecurity understanding	Communication gaps	**There is a disconnect between groups that play key roles in biosecurity at competitions.**
Biosecurity not hinderance if there is financial success	Financial considerations	Biosecurity motivations	**Biosecurity at competitions is a balancing act among other important considerations.**
Biosecurity processes exceeding expectations	Not a challenge	Biosecurity motivations	**Biosecurity at competitions is a balancing act among other important considerations.**
Can't control biosecurity at other competitions	Individualization	Personalized approach	**There is a disconnect between groups that play key roles in biosecurity at competitions.**
Challenge in implementation	Implementation challenges	Biosecurity drawbacks	**Biosecurity at competitions is a balancing act among other important considerations.**

Common sense when making biosecurity choices	Biosecurity understanding	Education	**There is a disconnect between groups that play key roles in biosecurity at competitions.**
Communication from organizers to competitors	Sharing information	Communication gap	**There is a disconnect between groups that play key roles in biosecurity at competitions.**
Comparison to other countries	Individualization	Communication gap	**There is a disconnect between groups that play key roles in biosecurity at competitions.**
Competitor responsibility in biosecurity	Who is responsible	Shared responsibility	**There is a disconnect between groups that play key roles in biosecurity at competitions.**
Braider responsibility	Who is responsible	Shared responsibility	**There is a disconnect between groups that play key roles in biosecurity at competitions.**
Governing body responsibility when it comes to biosecurity	Who is responsible	Shared responsibility	**There is a disconnect between groups that play key roles in biosecurity at competitions.**
Organizer's role in biosecurity	Who is responsible	Shared responsibility	**There is a disconnect between groups that play key roles in biosecurity at competitions.**
Role of the Venue in biosecurity	Who is responsible	Shared responsibility	**There is a disconnect between groups that play key roles in biosecurity at competitions.**
Trainer responsibility	Who is responsible	Shared responsibility	**There is a disconnect between groups that play key roles in biosecurity at competitions.**
Vet responsibility	Who is responsible	Shared responsibility	**There is a disconnect between groups that play key roles in biosecurity at competitions.**

COVID's role in biosecurity awareness	Biosecurity understanding	Biosecurity motivations	**Biosecurity at competitions is a balancing act among other important considerations.**
Additional Time	Time constraints	Biosecurity drawbacks	**Biosecurity at competitions is a balancing act among other important considerations.**
Compensation	Financial considerations	Biosecurity drawbacks	**Biosecurity at competitions is a balancing act among other important considerations.**
Costs to the venue	Financial considerations	Biosecurity drawbacks	**Biosecurity at competitions is a balancing act among other important considerations.**
Human Labour	Financial considerations	Biosecurity drawbacks	**Biosecurity at competitions is a balancing act among other important considerations.**
Organizer Costs	Financial considerations	Biosecurity drawbacks	**Biosecurity at competitions is a balancing act among other important considerations.**
Participant Cost	Financial considerations	Biosecurity drawbacks	**Biosecurity at competitions is a balancing act among other important considerations.**
Education to improve biosecurity	Education	Education	**There is a disconnect between groups that play key roles in biosecurity at competitions.**
Education in the form of communication of why it is important	Sharing information	Education	**There is a disconnect between groups that play key roles in biosecurity at competitions.**
Education on biosecurity coming from Coaches	Education	Education	**There is a disconnect between groups that play key roles in biosecurity at competitions.**

Organizer Education opportunities	Education	Education	**There is a disconnect between groups that play key roles in biosecurity at competitions.**
Enforcement of Rules	Implementation challenges	Biosecurity drawbacks	**Biosecurity at competitions is a balancing act among other important considerations.**
Fear of bringing diseases home from a show	Emotional toll	Biosecurity motivations	**Biosecurity at competitions is a balancing act among other important considerations.**
Financial concerns with following biosecurity	Financial considerations	Biosecurity drawbacks	**Biosecurity at competitions is a balancing act among other important considerations.**
Historical biosecurity requirements	Previous experience	Education	**There is a disconnect between groups that play key roles in biosecurity at competitions.**
Illegible verification documents	Implementation challenges	Biosecurity drawbacks	**Biosecurity at competitions is a balancing act among other important considerations.**
Increased awareness of biosecurity	Biosecurity understanding	Biosecurity motivations	**Biosecurity at competitions is a balancing act among other important considerations.**
Information given but not read	Implementation challenges	Communication gap	**There is a disconnect between groups that play key roles in biosecurity at competitions.**
Licensing requirements	Implementation challenges	Biosecurity drawbacks	**Biosecurity at competitions is a balancing act among other important considerations.**
More important than the importance it is given	Underrated	Biosecurity motivations	**Biosecurity at competitions is a balancing act among other important considerations.**

Other priorities over biosecurity	Implementation challenges	Biosecurity drawbacks	**Biosecurity at competitions is a balancing act among other important considerations.**
Outbreaks not widely shared	Sharing information	Communication gap	**There is a disconnect between groups that play key roles in biosecurity at competitions.**
Overdoing of biosecurity requirements at horseshows	Overrated	Biosecurity is low priority	**Biosecurity at competitions is a balancing act among other important considerations.**
Permission for high ranking officials at lower level events	Implementation challenges	Education	**There is a disconnect between groups that play key roles in biosecurity at competitions.**
Personal interest in biosecurity	Previous experience	Biosecurity motivations	**Biosecurity at competitions is a balancing act among other important considerations.**
Personal interest over the interest of the whole	Previous experience	Biosecurity motivations	**Biosecurity at competitions is a balancing act among other important considerations.**
Placing of blame should something happen	Who is responsible	Shared responsibility	**There is a disconnect between groups that play key roles in biosecurity at competitions.**
Safety of the horse	Care of the horse	Biosecurity motivations	**Biosecurity at competitions is a balancing act among other important considerations.**
Scientific advancement in infectious disease	Biosecurity understanding	Education	**There is a disconnect between groups that play key roles in biosecurity at competitions.**
Seeming lack of drug testing	Implementation challenges	Biosecurity drawbacks	**Biosecurity at competitions is a balancing act among other important considerations.**

Sense of ownership in design of biosecurity protocols	Individualization	Personalized approach	**There is a disconnect between groups that play key roles in biosecurity at competitions.**
Shared responsibility for biosecurity	Who is responsible	Shared responsibility	**There is a disconnect between groups that play key roles in biosecurity at competitions.**
Sharing of best practices	Sharing of information	Education	**There is a disconnect between groups that play key roles in biosecurity at competitions.**
Agricultural Associations	Source of information	Communication gap	**There is a disconnect between groups that play key roles in biosecurity at competitions.**
Equestrian professionals	Source of information	Communication gap	**There is a disconnect between groups that play key roles in biosecurity at competitions.**
Governing bodies	Source of information	Communication gap	**There is a disconnect between groups that play key roles in biosecurity at competitions.**
Social media	Source of information	Communication gap	**There is a disconnect between groups that play key roles in biosecurity at competitions.**
University	Source of information	Communication gap	**There is a disconnect between groups that play key roles in biosecurity at competitions.**
Veterinary Input	Source of information	Communication gap	**There is a disconnect between groups that play key roles in biosecurity at competitions.**
Turning a blind eye to horse health status	Poor horse care	Competition risk	**Biosecurity at competitions is a balancing act among other important considerations.**

Use of technology for increased efficiency	Future recommendations	Personalized approach	**There is a disconnect between groups that play key roles in biosecurity at competitions.**
Variability in biosecurity at different competitions	Individualization	Competition risk	**Biosecurity at competitions is a balancing act among other important considerations.**
How previous outbreak experience changes relationship to biosecurity	Previous experience	Biosecurity motivations	**Biosecurity at competitions is a balancing act among other important considerations.**
How Ship-in vs Stabling affects biosecurity	Individualization	Personalized approach	**There is a disconnect between groups that play key roles in biosecurity at competitions.**
Regional dependent biosecurity perspectives	Individualization	Personalized approach	**There is a disconnect between groups that play key roles in biosecurity at competitions.**
Show Duration and its effect on biosecurity	Individualization	Personalized approach	**There is a disconnect between groups that play key roles in biosecurity at competitions.**
Show Size and its effect on biosecurity	Individualization	Personalized approach	**There is a disconnect between groups that play key roles in biosecurity at competitions.**
Show Type and level and its effect on biosecurity	Individualization	Personalized approach	**There is a disconnect between groups that play key roles in biosecurity at competitions.**
